# Supplementary material for: A Narrative Review of Antibiotic Prescribing Practices in Primary Care Settings in South Africa and Potential Ways Forward to Reduce Antimicrobial Resistance
Source: Antibiotics (Basel). 2023 Oct 14;12(10):1540. doi: 10.3390/antibiotics12101540 (PMC10604704; doi:10.3390/antibiotics12101540)
Supplement: Supplementary file 1 [file antibiotics-12-01540-s001.zip › antibiotics-2616128-supplementary.pdf]

Supplementary materials

Table S1. Suggested activities in the short to medium term to reduce inappropriate prescribing of antibiotics in primary care settings in South Africa.

**Key Groups**	**Suggested Activities**
	**A) Prescribers**
**Ministry of Health, Medical Aid Groups and Health Insurance Groups**	• Work closely with HCPs in both the private and public primary healthcare sectors to routinely monitor prescribing as part of the National Action Plan to reduce AMR across South Africa [15], given concerns with current prescribing patterns (Tables 2 and 3). This includes gaining a better understanding of current utilisation patterns with the help of specially developed and easy-to-use electronic applications (Apps)
	• Work closely with leading HCPs and their organisations to refine and agree on future quality/prescribing indicators to use to improve future antibiotic prescribing in primary healthcare across the sectors, including adherence to agreed-upon guidelines given concerns. Existing prescribing/quality indicators (Table 5) can be used as a starting point. However, care should be taken not to overload prescribers with too many indicators to monitor
	• Agreed-upon indicators can subsequently be tested in practice among primary healthcare HCPs, especially those in the public health system in a testing protocol, and refined before full roll-out
	• Where appropriate, seek to refine current treatment guidelines based on the AWaRe book (which offers prescribing guidance for the vast majority of infectious diseases seen in ambulatory care in South Africa) [34,123]. Subsequently, instigate training sessions for key HCP groups, if needed, surrounding the AWaRe book and guidance to enhance adherence given current variable adherence to guidelines across sectors (Tables 2 and 3). Training sessions are increasingly likely to involve hybrid learning, building on the experiences during the COVID-19 pandemic [155]
	• Key targets for ASPs with HCPs under the NAP include reducing the prescribing of antibiotics for essentially viral infections based on an assessment of risk for the patients, including those with other infections, including HIV and TB, given high prevalence rates in South Africa [100]. As part of this, continue to evaluate point-of-care testing possibilities across sectors to improve future antibiotic prescribing [69,106]
	• The frequency and objectivity of monitoring activities as part of agreed-upon indicators and ASPs will be enhanced through the implementation of electronic healthcare records rather than current paper-based systems. The instigation of Apps that can quickly record consultation activities, including the prescribing of antibiotics and the indication thereof, is another alternative
	• As part of these activities, prescribers should be fully aware of the appropriate diagnostic codes to use. Activities can potentially include instigating training sessions for prescribers where appropriate
	• Alongside these measures, prescribers should work with pharmacists and others in PHCs and CHCs to reduce any shortages of key antibiotics to reduce the likelihood of being prescribed/dispensed inappropriate antibiotics for given infections. Recent improvements in supply chain management in the public healthcare system in South Africa should assist with this [156]
	• Multidisciplinary collaboration is essential across sectors to improve future antibiotic prescribing. The Ministry of Health, including relevant Ministerial Advisory Committees and managed care staff from private insurance companies/medical aid groups, are a key element alongside HCPs alongside current patient advocacy groups in South Africa
	**B) Patients**
	• Existing communication channels with patients should be improved to improve their knowledge of antibiotics and AMR, given current concerns, including pressure on HCPs to inappropriately prescribe antibiotics. This includes addressing current knowledge gaps regarding limited effectiveness of antibiotics for essentially viral infections, including ARIs, coupled with concerns with their overuse enhancing adverse reactions and AMR. Inputs could be via targeted educational campaigns in both urban and rural areas
	• As part of effective educational campaigns, government/private health insurance personnel should ensure that patients are familiar with the meaning of any terms used, including antibiotics, AMS and AMR, given current concerns [104]. This should include pre-testing educational campaigns beforehand for their understanding of advocacy and other groups
	• Potential channels for effective communication with patients increasingly include social media outlets, given their increasing impact on influencing patient behaviour [131], along with more traditional methods, including posters in HCP surgeries/facilities and pharmacies

	**C) Universities**
	• Universities should make sure HCPs are fully aware of the core concepts surrounding the use of antibiotics, including the AWaRe classification and guidelines [93,123], AMS and ASPs, by critically examining current curricula for undergraduate training as well as post-qualification educational input via continuous professional development (CPD) activities<br>• Activities also include making sure HCPs are equipped during their training with the necessary knowledge and skills for appropriate antibiotic prescribing and promoting AMR awareness so they are ready to start prescribing and advising patients post qualification<br>• Universities need to make sure that educational activities are still undertaken post qualification as part of CPD activities, given current concerns and the need to drive down AMR<br>• Private insurers and the Ministry of Health personnel need to work with key personnel in universities to research and monitor current prescribing practices across sectors, including their prescribing against agreed-upon indicators<br>• Private insurers and the Ministry of Health need to work with universities to instigate potential interventions (ASPs) based on agreed-upon indicators. Subsequently, these should be introduced, and their impact should be monitored. Following this, the ASPs should be refined if needed, i.e., if they are not achieving their desired impact (Table 6)
**HCP Associations dealing with primary healthcare settings**	• Work with the Ministry of Health and universities to design optimal communication strategies to limit patients' expectations that they will be dispensed an antibiotic for essentially self-limiting conditions<br>• As part of this, take part in ongoing CPD activities with ambulatory care prescribers, including surrounding the 'AWaRe' book [34,153] and its implications, to optimise the care of patients with infectious diseases in the community and reduce subsequent AMR<br>• Work with the Ministry of Health and private insurer personnel to introduce, test and monitor pertinent prescribing/quality indicators to improve appropriate prescribing of antibiotics across the sectors. As part of this, systems that can better monitor antibiotic utilisation should be introduced, including the instigation of specific Apps to monitor care—this is imperative as part of the development and instigation of appropriate prescribing/quality indicators<br>• Work with the Ministry of Health and others to reduce potential shortages of medicines among public health clinics (PHCs and CHCs)—especially if this means necessarily dispensing 'Watch' vs. 'Access' antibiotics<br>• Leverage the needs and opportunities to provide relevant CPD, AMR education and antibiotic guidelines to key prescribers. Apps surrounding the AWaRe guidance can increasingly be used to provide high-impact (and cost-effective) advice and guidance<br>• Work with Ministry of Health and private insurance personnel concerning effective ways to improve the education of patients to reduce inappropriate requests for antibiotics, especially for essentially self-limiting conditions
**HCP Associations (Generally) and Professional Councils**	• Work with the Ministry of Health and universities to design optimal communication strategies to limit patients' expectations that they will be dispensed an antibiotic for essentially self-limiting conditions<br>• As part of this, take part in ongoing CPD activities with ambulatory care prescribers, including surrounding the 'AWaRe' book [34,153] and its implications, to optimise the care of patients with infectious diseases in the community and reduce subsequent AMR<br>• Work with Ministry of Health and private health insurers to introduce, test and monitor pertinent prescribing/quality indicators to improve appropriate prescribing of antibiotics across sectors. As part of this, seek to introduce systems that can better monitor antibiotic utilisation, including the instigation of specific Apps to monitor care—this is imperative as part of the development and instigation of appropriate prescribing/quality indicators<br>• Work with the Ministry of Health and others to reduce potential shortages of medicines among public health clinics (PHCs and CHCs), especially if this means necessarily dispensing 'Watch' vs. 'Access' antibiotics<br>• Leverage the needs and opportunities to provide relevant CPD, AMR education and antibiotic guidelines to key prescribers. Apps surrounding the AWaRe guidance can increasingly be used to provide high-impact (and cost-effective) advice and guidance<br>• Work with Ministry of Health and key private insurance/medical aid personnel concerning effective ways to improve the education of patients in order to reduce inappropriate requests for antibiotics, especially for essentially self-limiting conditions
**Patients and Patient Associations,**	• Work with all key stakeholder groups to ensure that any messaging to patients is understandable given the extent of misconceptions currently regarding antibiotics and their use as well as AMR given current concerns (Table 4)

**including Advocacy Groups**	• Subsequently, work with health authorities and others to use social media to rapidly spread key messages regarding antibiotics and their use, as well as AMR, in a language that is understandable among patients<br>• Work with the Ministry of Health, private health insurance/medical aid personnel and others to address key misconceptions currently circulating with respect to antibiotics and their uses as well as AMR, especially for essentially self-limiting conditions<br>• Work with key groups, including university personnel, to enhance the communication skills of key prescribers, especially surrounding infectious diseases likely to be seen in primary health care settings, as well as misconceptions surrounding antibiotics and AMR<br>• Work with the Ministry of Health, private health insurance and others regarding the development of pertinent future dispensing or quality indicators
**Pharmaceutical companies**	• Promote responsible behaviour in the marketing of antibiotics in South Africa, especially if there are concerns<br>• This includes any communication with patients via social media and other platforms

NB: ARI = acute respiratory Illness; AMR = antimicrobial resistance; ASPs = Antimicrobial Stewardship Programmes; AWaRe classification [93,94]; CPD = continuous professional development; HCPs = healthcare professionals; LMICs = low- and middle-income countries; URTI: upper respiratory tract infection.

Table S2. ASP activities introduced across LMICs to improve antimicrobial prescribing in ambulatory care and their impact.

Country, Author and Year	Intervention	Key Findings and Impact
China, Yip et al., 2014 [157]	• Principally involving financial incentives<br>• Randomised study undertaken to evaluate the impact of capitation with the introduction of pay-for-performance measures on subsequent antibiotic prescribing, health spending, outpatient visit volumes and patient satisfaction in Ningxia Province	• There was approximately 15% reduction in antibiotic prescriptions following the implementation of pay-for-performance measures<br>• A small reduction in total spending per visit was also seen
China, Zhang et al, 2018 [158]	• The objective of the study was to evaluate the cost-effectiveness of a health behaviour change intervention (information and leaflets were given to parents/caregivers) to reduce antibiotic prescribing<br>• The primary endpoint of the study was to lower the proportion of prescriptions containing at least one antibiotic given to children with URTIs and the cost-effectiveness associated with any reduction in the prescribing of antibiotics<br>• Costs included the average duration of consultations multiplied by the cost of the doctor's time alongside the cost of the leaflets, medicine and implementation costs	• There was an appreciable reduction (29%) in the prescribing of antibiotics in the active versus control arms ($p = 0.0002$)<br>• There was an incremental cost of $1.02 per patient in the intervention arm compared with the control arm<br>• However, when taking into account the cost of medicines, etc., the incremental cost was $0.03/% point reduction in antibiotic use, which is close to cost-neutral
Lao, Haenssgen et al., 2018 [159]	• Implementation of multifaceted educational activities in two Lao villages to share general antibiotic-related messages<br>• Alongside this, learn about people's conceptions regarding antibiotics and AMR as well as health behaviours	• Overall mixed findings:<br>o The average recognition of the term for drug resistance rose from 27.6% to 91.4% among the educated group compared to 36.2% to 58.8% among the control group<br>o However, the impact of the educational activities on subsequent antibiotic use was inconclusive
China, Wei et al., 2019 [160]	• Principally education involving multiple interventions<br>• Interventions comprised the following:<br>o Clinical guidelines<br>o Monthly prescribing review meetings<br>o Training in doctor–patient communication skills	• A 49% reduction in the prescribing of antibiotics for children with URTIs after 6 months in the intervention arm—having adjusted for patient and prescribing doctor covariates<br>• The reductions persisted after 18 months but at a lower rate (-36%)<br>• Key factors for sustaining the reduction in antibiotic prescribing included physicians' improved

	• Provision of education materials for caregivers	knowledge and communication skills combined with prescription review meetings
India, Dehn Lunn, 2018 [161]	• Principal education to reduce the rate of inappropriate prescribing for URTIs<br>• Education consisted of a repeated process of audit and feedback combined with interactive training sessions, one-to-one case-based discussion, antibiotic guideline development and coding updates	• Antibiotic prescribing reduced from 62.6% of patients prescribed antibiotics to just 7.2% following the intervention<br>• This was combined with an increase in the documentation of examination findings from 52.7% to 95.6% of patients justifying the treatment approach
Kenya, Egger et al., 2017 [162]	• Comprehensive set of interventions was used to improve the management of 4 common conditions in PHCs: URTIs, UTIs, STIs and childhood diarrhoea<br>• Interventions included the following:<br>o Online educational programmes explaining the clinical guidelines for these 4 conditions<br>o Two-hour educational sessions<br>o Monthly feedback meetings<br>• Materials included posters and other materials to remind prescribers	• Adherence to agreed-upon clinical quality measures (CQM) increased from 41.4% to 77.1% for PHCs that took part in the intervention<br>• However, adherence dropped slightly from 26.5% to 21.8% among control PHCs over the 6-month study period. This was greatest for UTIs<br>• Adherence to agreed clinical quality measures significantly increased over the 6-month study period for the active intervention group<br>• The interventions were well received by those operating in the PHCs
Kenya, Korom et al., 2017 [163]	• Multiple interventions principally surrounding education. These included the following:<br>o Formal introduction of a clinical practice guideline<br>o Introduction of peer-to-peer chart reviews<br>o Peer-reviewed publication describing local AMR patterns<br>• Interventions were undertaken by trained clinical officers	• Adherence to guideline-recommended antibiotics improved significantly, i.e. from 19% at baseline to 68% following all interventions (X2 = 150.7, $p < 0.001$)<br>• An outcome of composite quality scores improved significantly from an average of 2.16 to 3.00 on a 5-point scale (t = 6.58, $p < 0.001$)<br>• The interventions had different effects at different clinical sites—possibly reflecting differences in clinical officers and their activities<br>• Provider age was not a significant factor in subsequent changes in prescribing habits
Kenya, Kleczka et al., 2019 [164]	• Multiple components to this ASP among PHCs in the private sector in Kenya. Interventions included the following:<br>o Rubber stamp templates for documenting the management of selected conditions, e.g., URTIs, UTIs, STIs and GIs<br>o Compilations of the relevant clinical practice guidelines for discussion/adherence<br>o One low-budget Android smartphone to each facility<br>o Six continuing medical education (CME) sessions at each facility every month for 6 months to improve compliance<br>• Adherence to guidelines was determined using two measures—these included the appropriateness of the diagnosis and the appropriateness of prescribing based on current guidelines	• Antibiotics were prescribed in 94.3% ± 1.6% of the 889 patient encounters documented with templates including 97.3% ± 2.3% for URTI encounters, 94.2% ± 3.8% for UTI encounters, 91.6%±1.1% for STI encounters and 91.3% ± 1.4% for GI encounters<br>• Overall template documentation scores (69.5% ± 1.7%) were significantly higher post interventions<br>• The prescribing of nitrofurantoin in patient encounters increased from 9.2% to 29.9%; $p < 0.0001$<br>• The prescribing of broad-spectrum quinolones (ciprofloxacin—**W;** norfloxacin—**W**) decreased from 30.0% of encounters to just 16.1% ($p < 0.05$)
Malaysia, Tay et al., 2019 [165]	• Principal education involving educational toolkits, which included the following:<br>o The production of a training module for HCPs on ARIs and acute diarrhoea<br>o One-hour educational sessions covering diagnostic criteria and treatment decision pathways<br>o Educational posters in Malay and English in the waiting area and consultation rooms	• Appreciable reduction in antibiotic prescribing:<br>o Prescribing of antibiotics for ARIs down from 29.1% to 13.7% of patients<br>o Prescribing of antibiotics for acute diarrhoea down from 11.2% to 6.7%

	o Multimedia educational videos in the waiting areas<br>o Physician reminders	
Namibia, Brinkmann et al., 2020 [166]	• Activities included assessing the effectiveness of implementation of ASPs among 10 PHCs in Namibia<br>• SWOT analysis of each health facility was conducted by interviewing infection control focal personnel at each facility to assess the level of compliance with good AMS practices and policies	• 90% of the focal persons were aware of systems and policies for good AMS practices<br>• The level of compliance at hospital-based PHCs was 30.8% compared to clinics (9.1% to 36.4%)<br>• Principal challenges to implementing ASPs in Namibia included the following:<br>o Lack of policies and systems specific to antimicrobial use<br>o Financial and human resource concerns
Sierra Leone, Hamilton et al., 2018 [167]	• Principal educational initiatives in the ASP<br>• Provision of an empirical antimicrobial guideline introduced via a number of different methods, which included the following:<br>o One-to-one feedback meetings with prescribers<br>o Announcements of the guidelines in general meetings<br>• Printed copies of the guideline in each outpatient room to help educate patients	• After the first cycle, the choice of appropriate antimicrobial in prescriptions improved to 85%, and the correct antibiotic, dose and course length improved to 53%<br>• Unfortunately, after 2 months, the rates of appropriate prescribing reduced to 65% and 43%, respectively, following a lack of follow-up<br>• Overall, implementing guidelines can be effective in improving appropriate antibiotic prescribing in ambulatory care; however, repeated measures are needed for sustainable changes
Tanzania, Rambaud-Althaus et al., 2017 [168]	• Principal education with 48 health workers (18 paper-based and 30 in the electronic arm) involved in treating childhood infections receiving a 2-day training session through lectures and practical exercises on AMR and guideline management of target diseases<br>• On the second day (in the afternoon), the health workers were trained separately on the use of guidelines with clinical case studies (either via booklets or smartphone applications among those in the electronic arm)	• A total of 504 consultations were observed 4 months after the training<br>• The use of smartphones vs. paper-based guidelines/education resulted in a significant increase in children checked for danger signs—these included difficulties with drinking, vomiting and convulsions (41% versus 74%, $p = 0.04$).<br>• The rate of antibiotic prescriptions for childhood infections dropped from 70% among children being treated for the target diseases in the control to 26% in the paper-based arm and 25% in the electronic arm<br>• The proportion of children not needing antibiotics and for whom they were not prescribed was higher in the paper-based (75%) and electronic arms (80%) than in the control arm
Thailand, Boonyasiri et al., 2014 [169]	Principal educational initiatives included the following:<br>• Training HCPs on the rational use of antibiotics<br>• Introducing clinical practice guidelines with the potential for throat swabs (ARIs) and stool cultures (acute diarrhoea)<br>• Printed brochures for patients/relatives in waiting rooms containing likely aetiology of the principal conditions as well the potential necessity/harm of antibiotics for ARIs and acute diarrhoea	• The multifaceted programme resulted in the following:<br>o Limited prescribing of antibiotics for ARIs (13.0%) and for acute diarrhoea (19.1%)<br>o Clinical responses on day 3 after receiving care from the HCPs showed that more than 97% of the patients who received antibiotics/those who did not receive antibiotics were cured or improved, confirming the soundness of the approach
Vietnam, Hoa et al., 2017 [170]	• Multifaceted educational intervention over 7 months targeting HCPs' knowledge, practical competencies and prescribing of antibiotics for patients with ARI in the intervention group<br>• There are no specific programmes in the control group	• Knowledge regarding antibiotics improved in the intervention group for patients with ARIs by 28%, antibiotic use for mild ARIs by 15% and severe ARIs by 14%<br>• Practical competence for patients with mild ARIs improved in the intervention and control groups by 20% and 11%, respectively

		• Practice regarding the prescribing of antibiotics for mild ARIs improved by 28% in the intervention group

NB: ARI = acute respiratory illness; AMS = Antimicrobial Stewardship; ASP = Antimicrobial Stewardship Programme; HCP = Healthcare Professional; STIs = Sexually Transmitted Infections; URTIs = Upper Respiratory Tract Infections ; UTIs = Urinary Tract Infections
